# The relationship between visceral obesity and hepatic steatosis measured by controlled attenuation parameter

**DOI:** 10.1371/journal.pone.0187066

**Published:** 2017-10-27

**Authors:** Hye Won Lee, Kwang Joon Kim, Kyu Sik Jung, Young Eun Chon, Ji Hye Huh, Kyeong Hye Park, Jae Bock Chung, Chang Oh Kim, Kwang-Hyub Han, Jun Yong Park

**Affiliations:** 1 Department of Internal Medicine, Institute of Gastroenterology, Yonsei University College of Medicine, Seoul, Republic of Korea; 2 Yonsei Liver Center, Severance Hospital, Seoul, Republic of Korea; 3 Division of Geriatrics, Department of Internal Medicine, Yonsei University College of Medicine, Seoul, Republic of Korea; 4 Department of Internal Medicine, CHA Bundang Medical Center, CHA University, Seongman, Republic of Korea; 5 Division of Endocrinology and Metabolism, Department of Internal Medicine, Yonsei University, Wonju College of Medicine, Wonju, Republic of Korea; 6 Division of Endocrinology and Metabolism, Department of Internal Medicine, National Health Insurance Service Ilsan Hospital, Goyang, Gyeonggi, Republic of Korea; Taipei Veterans General Hospital, TAIWAN

## Abstract

**Background:**

Nonalcoholic fatty liver disease (NAFLD) is closely related with obesity. However, obese subjects, generally represented by high BMI, do not always develop NAFLD. A number of possible causes of NAFLD have been studied, but the exact mechanism has not yet been elucidated.

**Methods:**

A total of 304 consecutive subjects who underwent general health examinations including abdominal ultrasonography, transient elastography and abdominal fat computed tomography were prospectively enrolled. Significant steatosis was diagnosed by ultrasonography and controlled attenuation parameter (CAP) assessed by transient elastography.

**Results:**

Visceral fat area (VFA) was significantly related to hepatic steatosis assessed by CAP, whereas body mass index (BMI) was related to CAP only in univariate analysis. In multiple logistic regression analysis, VFA (odds ratio [OR], 1.010; 95% confidence interval [CI], 1.001–1.019; *P* = 0.028) and triglycerides (TG) (OR, 1.006; 95% CI, 1.001–1.011; *P* = 0.022) were independent risk factors for significant hepatic steatosis. The risk of significant hepatic steatosis was higher in patients with higher VFA: the OR was 4.838 (*P*<0.001; 95% CI, 2.912–8.039) for 100<VFA≤200 cm^2^ and 7.474 (*P*<0.001; 95% CI, 2.462–22.693) for VFA >200 cm^2^, compared to patients with a VFA ≤100 cm^2^.

**Conclusions:**

Our data demonstrated that VFA and TG is significantly related to hepatic steatosis assessed by CAP not BMI. This finding suggests that surveillance for subjects with NAFLD should incorporate an indicator of visceral obesity, and not simply rely on BMI.

## Introduction

Nonalcoholic fatty liver disease (NAFLD) is one of the most common causes of chronic liver disease worldwide. Severe forms of NAFLD such as nonalcoholic steatohepatitis can progress to end-stage liver disease such as cirrhosis or hepatocellular carcinoma [1–3]. Therefore, investigating risk factors associated with hepatic steatosis is required to perform effective screening. Hepatic steatosis develops for a variety reasons, but obesity is the most common associated condition [4] and vice versa. Cuf-off of body mass index (BMI) defining obesity differs according to race. The recommended BMI cut-off value for obesity is > 25 kg/m^2^ for Asians, in contrast to > 30 kg/m^2^ for Western individuals [5]. However, NAFLD can occur in non-obese subjects and NAFLD in non-obese patient is especially frequent in Asia [6].

Liver biopsy is the gold standard for diagnosing fatty liver disease, but it is invasive and difficult to perform in a clinical setting. As a non-invasive method, transient elastography (TE) has been validated for assessing hepatic steatosis using a controlled attenuation parameter (CAP) [7]. In a recent study, the CAP score and liver stiffness values assessed by TE showed significant correlation with the degrees of steatosis (r = 0.656, *P*<0.001) and fibrosis (r = 0.714, P<0.001) [8]. A meta-analysis also showed that CAP provides a standardized non-invasive measurement of hepatic steatosis [9].

Several studies suggested visceral adiposity to be a clinical predictor of hepatic steatosis [10–12]. Visceral fat area (VFA) measured by computed tomography (CT) is a quantitative and accurate measure of visceral fat, which is more predictive of hepatic steatosis than BMI or subcutaneous fat accumulation [13,14]. Although previous studies have identified a relationship between VFA and histological hepatic steatosis, the relationship between VFA and steatosis is not widely used in clinical practice due to the invasiveness of liver biopsy [15]. To facilitate clinical application, the relationship between VFA and non-invasive methods of assessing hepatic steatosis should be evaluated.

As a non-invasive and relatively accurate method of quantifying hepatic steatosis, CAP has other advantages, such as operator independency, and lower costs, when compared to other non-invasive methods such as ultrasonography, CT, and magnetic resonance imaging (MRI) [16–20]. However, the relationship between CAP and clinical factors, such as indicators of visceral adiposity, is still unclear. The aim of this study was to evaluate the relationship between CAP and VFA (quantitative indicators of hepatic steatosis and central obesity, respectively), together with other clinical factors.

## Materials

### Patients

Between November 2011 and July 2012, 316 patients who underwent a general health examination that included abdominal ultrasonography, TE, and abdominal fat CT scan at Severance Hospital, Yonsei University College of Medicine, Seoul, Korea, were enrolled. Patients were excluded if they chronically consumed excessive alcohol (>30 g/day for men, >20 g/day for women) or were positive for serum hepatitis B surface antigen, or serum hepatitis C virus antibody. We also excluded patients in whom CAP measurements were unsuccessful. Finally, 304 patients were included in the statistical analysis. Written informed consent was obtained from all patients before enrollment. The study protocol conformed to the ethical guidelines of the 1975 Helsinki Declaration and was approved by the Institutional Review Board of Severance Hospital.

### Anthropometric data and laboratory tests

Clinical data and previous medical history were obtained by self-report questionnaires and an electronic chart review. Anthropometric measurements, including BMI, and waist-hip ratio (WHR), were performed on the same day as the laboratory and radiological tests. Body weight and height were measured using a digital scale, and BMI was calculated by dividing weight (kg) by the square of height (m^2^). Using a tape measure, a well-trained individual measured the waist circumference at the midpoint between the lower costal margin and anterior superior iliac crest, and the hip circumference at the widest point over the buttocks. WHR was obtained by dividing the mean waist circumference by the mean hip-circumference.

Laboratory parameters including serum fasting glucose, total cholesterol, triglycerides, aspartate aminotransferase (AST), alanine aminotransferase (ALT), γ-glutamyl transpeptidase (γ-GTP) and erythrocyte sedimentation rate (ESR) were measured on the same day as the radiological tests.

### Ultrasonographic assessments and measurement of controlled attenuation parameter

After fasting for at least 8 hours, all patients underwent abdominal ultrasonography and TE using the liver FibroScan^®^ (Echosens, Paris, France) M probe. Ultrasonographic examinations of the liver were performed by experienced radiologists who were blinded to the clinical information. Diagnosis of a fatty liver was performed by ultrasonography using previously described standardized criteria [21].

One experienced technician performed TE blinded to the clinical data of the patients’. The principles of CAP measurement have been described previously [16]. CAP measures ultrasonic attenuation at 3.5 MHz using signals acquired by TE. The interquartile range (IQR) was defined as an index of the intrinsic variability of CAP values corresponding to the interval of CAP results containing 50% of the valid measurements between the 25th and 75th percentiles. The median of successful measurements was selected as representative of the CAP values of a given patient. As an indicator of variability, the ratio of the IQR of CAP values to the median (IQR/M_CAP_) was calculated. In this study, only TE measurements with ≥ 10 valid shots, and a success rate of ≥ 60% were considered reliable and used for statistical analysis.

Steatosis (≥ TE based steatosis grade 1), was defined as the presence of fatty liver disease by abdominal ultrasonography findings and a CAP value ≥ 248 dB/m. Significant steatosis (≥ TE based steatosis grade 2) was defined as the presence of fatty liver disease on the images and a CAP value ≥ 268 dB/m [9].

### Assessment of abdominal visceral fat area and subcutaneous fat area

Subcutaneous and visceral fat areas were calculated by CT (Somatom Plus, Siemens, Germany). A lead protection device was used to minimize exposure to X-rays during CT scans. Subjects were examined in a supine position. The visceral and subcutaneous adipose regions were calculated according to the intervertebral position of L2–3. VFA was defined as intra-abdominal fat bound by the parietal peritoneum or transversals fascia, excluding the vertebral column and the paraspinal muscles. Subcutaneous fat area (SFA) was defined as fat superficial to the abdominal and back muscles. VAT was then measured around the inner boundary of the abdominal wall muscles. A region of interest drawn around the external margin of the dermis was used to calculate the total adipose tissue (TAT) area. The SFA was obtained by subtracting the VAT from the TAT.

### Statistical analysis

Data are expressed as mean ± standard deviation, median (range), or number (%), as appropriate. Correlations between CAP and other variables were described using Spearman’s correlation coefficients. Comparisons between patients with and without hepatic steatosis were performed using the Student’s *t*-test or the Mann-Whitney test for continuous variables, and the chi-squared or Fisher’s exact test was used for categorical variables. Univariate and subsequent multivariate binary logistic regression analyses were performed to identify independent factors related to significant hepatic steatosis. Odds ratio (OR) and corresponding 95% confidence interval (CI) were also evaluated. Optimal cut-off VFA values to predict significant hepatic steatosis were calculated as the maximized sum of the sensitivity and specificity (Youden index) from the areas under the receiver operating characteristic curves (AUROC). Positive predictive value and negative predictive value (PPV and NPV) were also computed. A *P* value <0.05 by two-tailed test was considered indicative of statistical significance. Data analyses were performed using the SAS software (ver. 9.1; SAS Inc., Cary, NC, USA).

## Results

### Baseline characteristics

Baseline characteristics of the study population are summarized in Table 1. The total number of subjects was 304 (165 men and 139 females) and their mean age was 56.5 ± 10.7 years. The mean BMI was 24.1 ± 3.1 kg/m^2^, and the WHR was 0.89 ± 0.04. Eighty (26.3%) patients were overweight (BMI, 23–25 kg/m^2^) and 114 (37.5%) were obese (BMI >25 kg/m^2^) according to the new World Health Organization BMI criteria for Asians.[22] The mean VFA and SFA were 111.4 ± 50.6 cm^2^ and 175.3 ± 60.0 cm^2^, respectively. The median CAP value was 244 dB/m (range, 100–382).

**Table 1:** Baseline characteristics (n = 304).

Variables	Total (n = 304)	Male group (n = 165, 54.3%)	Female group (n = 139, 45.7%)	*P*-value
Age, years	56.5 ± 10.7	56.6 ± 10.9	56.3 ± 10.6	NS
Medical history				
Diabetes mellitus	22 (7.2)	16 (9.7)	6 (4.3)	NS
Hypertension	44 (14.5)	29 (17.6)	15 (10.8)	NS
Body mass index, kg/m^2^	24.1± 3.1	25.1 ± 3.0	22.9 ± 2.7	<0.001
Waist/Hip ratio	0.89 ± 0.04	0.89 ± 0.04	0.86 ± 0.04	<0.001
Visceral fat area, cm^2^	111.4±50.6	123.5 ± 53.7	97.1 ± 42.6	<0.001
Subcutaneous fat area, cm^2^	175.3 ± 60.0	167.3 ± 60.3	184.8 ± 58.5	0.011
Laboratory profiles				
Fasting glucose. mg/mL	99.7 ± 24.2	104.6 ± 27.8	93.9 ± 17.4	<0.001
Total cholesterol, mg/mL	184.5 ± 37.8	178.0 ± 39.1	192.1 ± 34.7	0.001
Triglycerides. mg/mL	114.9 ± 63.7	128.6 ± 74.3	98.8 ± 43.2	<0.001
AST, IU/L	22.9 ± 9.0	24.3 ± 8.8	21.4 ± 7.9	0.003
ALT, IU/L	23.5 ± 13.5	26.6 ± 14.7	19.1 ± 10.5	<0.001
γ-GTP, IU/L	33.2 ± 30.9	42.7 ± 36.4	22.1 ± 17.3	0.001
ESR, mm/h	17.2 ± 15.5	13.4 ± 13.3	21.9 ± 16.8	<0.001
CAP (dB/m)	244 (100–382)	254 (127–367)	235 (100–382)	0.001
IQR (dB/m)	28.0 (7.0–75.0)	26.0 (7.0–65.0)	29 (8.0–75.0)	NS
IQR/Mcap	0.12 (0.02–0.25)	0.11 (0.02–0.21)	0.12 (0.02–0.25)	NS

Variables are expressed as mean ± standard deviation, median (range), or number (%). NS, not significant (P>0.05); AST, aspartate aminotransferase; ALT, alanine aminotransferase; γ-GTP, γ-glutamyl transpeptidase; ESR, erythrocyte sedimentation rate; CAP, controlled attenuation parameter; IQR, interquartile range

BMI, WHR, VFA, CAP value, and the serum levels of fasting glucose, triglycerides, AST, ALT, γ-GTP, and ESR were higher in males, whereas SFA and the serum cholesterol level were higher in females (Table 1).

### Correlations between controlled attenuation parameter and clinical variables

In univariate analyses, CAP values were correlated with the male gender (ρ = 0.173, *P* = 0.002), BMI (ρ = 0.491, *P*<0.001), VFA (ρ = 0.497, *P*<0.001), SFA (ρ = 0.234, *P*<0.001), fasting glucose (ρ = 0.406, *P*<0.001), triglycerides (ρ = 0.352, *P*<0.001), ALT (ρ = 0.285, *P*<0.001), and γ -GTP (ρ = 0.374, *P*<0.001). In a subsequent multivariate regression analysis, CAP (*P* = 0.001) was independently associated with VFA, triglycerides (*P*<0.001) and ALT (*P* = 0.017) (Table 2). The correlation between CAP and VFA is shown in S1 Fig.

**Table 2:** Univariate and multivariate regression analyses of clinical factors associated with controlled attenuation parameter.

Variable	Univariate		Multivariate
	ρ	*P* value (ρ)	*P* value
Male gender	0.173	0.002	NS
Body mass index, kg/m^2^	0.491	<0.001	NS
Visceral fat area, cm^2^	0.497	<0.001	0.001
Subcutaneous fat area, cm^2^	0.234	<0.001	NS
Fasting glucose, mg/mL	0.406	<0.001	NS
Triglycerides, mg/mL	0.352	<0.001	<0.001
ALT, IU/L	0.285	<0.001	0.017
γ-GTP, IU/L	0.374	<0.001	NS

NS, not significant; ALT, alanine aminotransferase; γ-GTP, γ-glutamyl transpeptidase

### Comparison of the hepatic steatosis and non-hepatic steatosis groups

Significant hepatic steatosis was observed in 134 (44.1%) patients. Significant steatosis was present in 93 (69.4%) patients (68 in male and 25 in female) in the obese group and 41 (30.6%) patients (21 in male and 20 in female) in the non-obese group.

In univariate analysis, male gender (90 of 134 patients [67.2%] vs. 49 of 170 patients [28.8%], *P* = 0.005), BMI (25.5 ± 3.1 kg/m^2^ vs. 23.0 ± 2.6 kg/m^2^, *P*<0.001), WHR (0.90 ± 0.04 vs. 0.86 ± 0.04, *P* = 0.001), VFA (131.5 ± 53.7 cm^2^ vs. 95.6 ± 41.9 cm^2^, *P*<0.001), SFA (191.3 ± 60.8 vs. 162.7 ± 56.5, *P*<0.001), fasting glucose (104.8 ± 23.8 mg/mL vs. 95.7 ± 23.7 mg/mL, *P* = 0.001), triglycerides (133.8 ± 71.4 mg/mL vs. 100.1 ± 52.6 mg/mL, *P*<0.001), ALT (26.5 ± 16.1 IU/L vs. 20.5 ± 10.4 IU/L, *P*<0.001), and γ-GTP (37.5 ± 32.3 IU/L vs. 29.8 ± 29.5 IU/L, *P* = 0.031) were higher in patients with significant hepatic steatosis than in those without (Table 3). When the patients with and without significant hepatic steatosis were compared according to gender, BMI, WHR, VFA, SFA, and serum level of fasting glucose, triglycerides, ALT, and γ-GTP were significantly higher in both genders with significant hepatic steatosis (S1 Table).

**Table 3:** Comparison of patients with and without significant hepatic steatosis.

Variable	No significant-hepatic steatosis (n = 170, 55.9%)	Hepatic steatosis (n = 134, 44.1%)	*P* value
Age, years	55.5 ± 11.5	57.7 ± 9.7	NS
Male gender (%)	49 (36.6)	90 (52.9%)	0.005
Medical history			
Diabetes mellitus	7 (4.1)	15 (11.2)	NS
Hypertension	26 (15.3)	18 (13.4)	NS
Body mass index, kg/m^2^	23.0 ± 2.6	25.5 ± 3.1	<0.001
Waist/hip ratio	0.86 ± 0.04	0.90 ± 0.04	0.001
Visceral fat area, cm^2^	95.6 ± 41.9	131.5 ± 53.7	<0.001
Subcutaneous fat area, cm^2^	162.7 ± 56.5	191.3 ± 60.8	<0.001
Laboratory profiles			
Fasting glucose, mg/mL	95.7 ± 23.7	104.8 ± 23.8	0.001
Cholesterol, mg/mL	182.3 ± 39.6	187.4 ± 35.3	NS
Triglycerides, mg/mL	100.1 ± 52.6	133.8 ± 71.4	<0.001
AST, IU/L	21.9 ± 7.2	**24.3** ± 9.8	NS
ALT, IU/L	20.5 ± 10.4	26.5 ± 16.1	<0.001
γ-GTP, IU/L	29.8 ± 29.5	37.5 ± 32.3	0.031
ESR, mm/h	17.1 ± 15.1	17.6 ± 16.2	NS
Liver stiffness value, kPa	4.5 (2.1–21.8)	4.6 (2.8–14.3)	NS

Variables are expressed as mean ± standard deviation, median (range), or number (%).

NS, not significant; AST, aspartate aminotransferase; ALT, alanine aminotransferase; γ-GTP, γ-glutamyl transpeptidase; ESR, erythrocyte sedimentation rate

### Risk factors for significant hepatic steatosis

**Fig 1:**
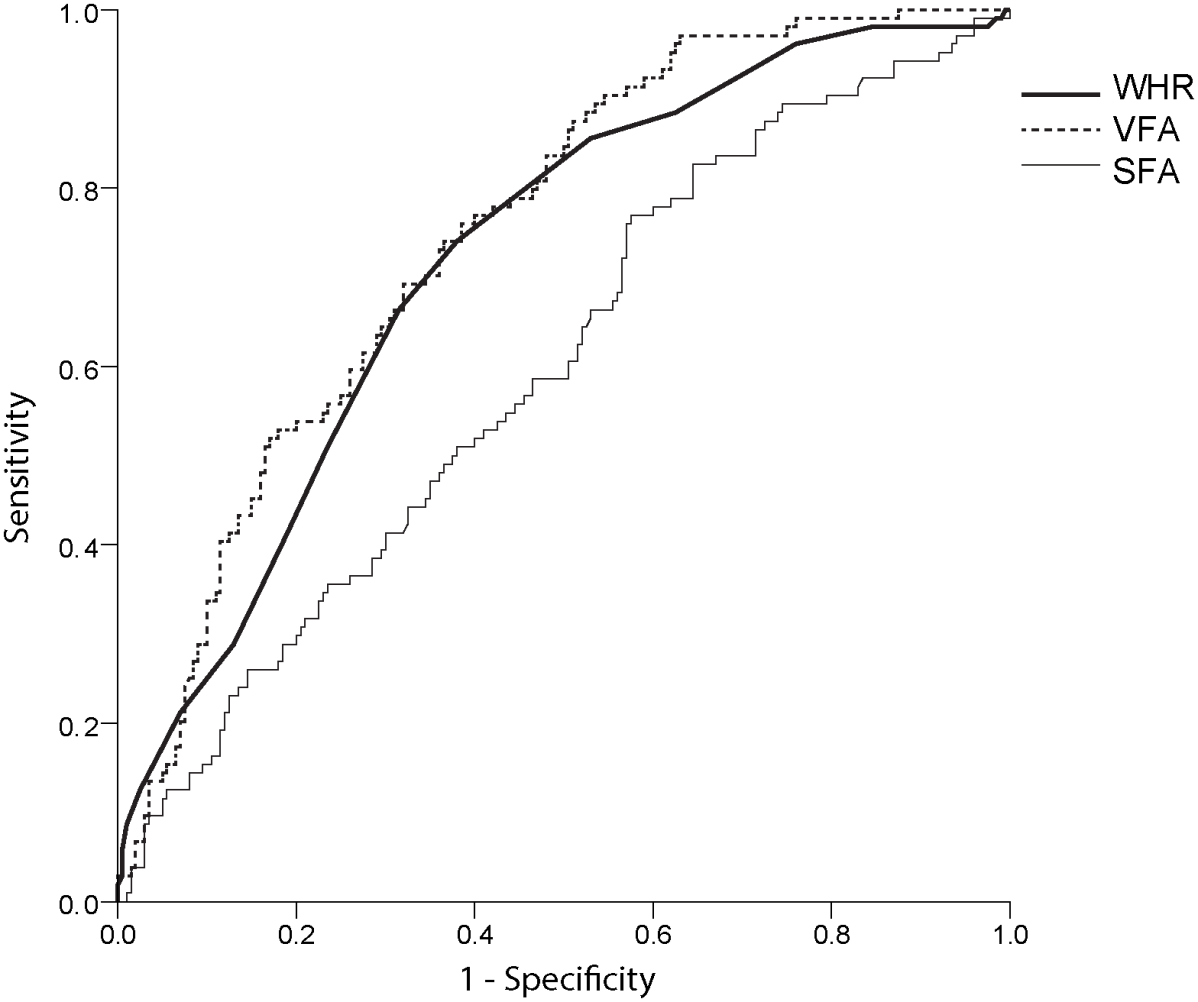
Areas under the receiver operating characteristic curves of the predictive power of hepatic steatosis.

In the multiple logistic regression analysis adjusted for age and gender, VFA (OR, 1.010; 95% CI, 1.001–1.019; *P* = 0.028) and triglycerides (OR, 1.006; 95% CI, 1.001–1.011; *P* = 0.022) were identified as independent risk factors for significant hepatic steatosis (Table 4). VFA exhibited a greater AUROC values than SFA and WHR (VFA, 0.750; SFA, 0.597; WHR, 0.721) (Fig 1). VFA (OR, 1.008; 95% CI, 1.001–1.011; *P* = 0.045) was the only independent risk factor for significant hepatic steatosis in males, whereas VFA (OR, 1.029; 95% CI, 1.010–1.048; P = 0.002), triglycerides (OR, 1.003; 95% CI, 1.006–1.026; *P* = 0.017), and ALT (OR, 1.057; 95% CI, 1.006–1.111; *P* = 0.029) were independent risk factors in females.

**Table 4:** Independent risk factors for significant hepatic steatosis.

Variable	Total (n = 304)		Male (n = 165, 54.3%)		Female (n = 139, 45.7%)	
	OR (95% CI)	*P* value	OR (95% CI)	*P* value	OR (95% CI)	*P* value
Male gender		NS				
Age		NS		NS		NS
BMI		NS		NS		NS
Visceral fat area	1.010 (1.001–1.019)	0.028	1.008 (1.001–1.011)	0.045	1.029 (1.010–1.048)	0.002
Subcutaneous fat area		NS		NS		NS
Fasting glucose		NS		NS		NS
Triglycerides	1.006 (1.001–1.011)	0.022		NS	1.003 (1.006–1.026)	0.017
ALT		NS		NS	1.057 (1.006–1.111)	0.029
γ-GTP		NS		NS		NS

OR, odds ratio; CI, confidence interval; NS, not significant; BMI, body mass index; ALT, alanine

#### Optimal cut-off visceral fat area values for predicting significant hepatic steatosis

The cut-off VFA value for predicting a low risk of significant hepatic steatosis with a high NPV (92.3%) was 57.3 cm^2^ (PPV, 47.5%; sensitivity 98.5%; specificity 14.1%). The cut-off value of VFA for predicting a high risk of significant hepatic steatosis with a high PPV (70.0%) was 192.2 cm^2^ (NPV 57.7%; sensitivity 10.4%; specificity 96.5%).

**Fig 2:**
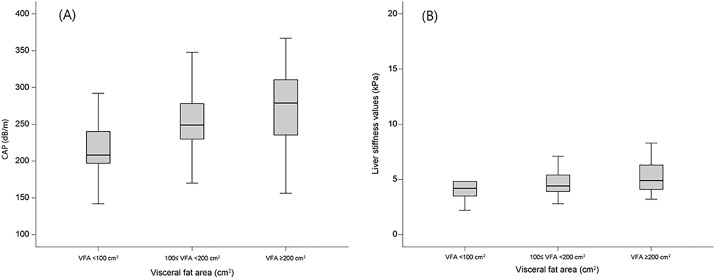
(A) Controlled attenuation parameter (CAP) score distribution in patients with visceral fat area (VFA) <100 cm^2^, 100≤VFA<200 cm^2^, and VFA ≥200 cm^2^. The median CAP score increased with increasing VFA. (B) Liver stiffness (LS) value distribution in patients with visceral fat area (VFA) <100 cm^2^, 100≤VFA<200 cm^2^, and VFA ≥200 cm^2^. The median LS value increased with increasing VFA.

When the population was stratified according to VFA <100 cm^2^, 100≤VFA<200 cm^2^, and VFA ≥200 cm^2^, patients with a higher VFA had a greater risk of significant hepatic steatosis. The ORs were 4.838 (*P*<0.001; 95% CI, 2.912–8.039) for patients with 100≤VFA<200 cm^2^ and 7.474 (*P*<0.001; 95% CI, 2.462–22.693) for those with a VFA ≥200 cm^2^, as compared to patients with a VFA <100 cm^2^. The median CAP score and LS value increased with increasing VFA (Fig 2A and 2B).

### Sub-group analysis of patients with a body mass index below 23 kg/m^2^

To identify risk factors for significant hepatic steatosis in non-obese patients, sub-group analysis of subjects with a BMI <23 kg/m^2^ was performed. Among 110 patients (76 males and 34 females), 22 (20.0%) had significant hepatic steatosis. In univariate analyses, VFA (*P*<0.001), glucose (*P* = 0.009), and triglycerides (*P* = 0.001) were associated with significant hepatic steatosis. In a subsequent multivariate analysis, only VFA was significantly related to significant hepatic steatosis (OR 1.006; 95% CI 1.001–1.011; *P* = 0.022).

## Discussion

NAFLD has become one of the most important and rapidly increasing diseases worldwide.[1] Metabolic syndrome, including obesity, has surprisingly become a major health problem in Asia in association with a changed in lifestyle. Our data suggest that VFA is significantly associated with hepatic steatosis, as assessed by TE. Patients with a higher VFA had a greater risk of significant hepatic steatosis compared with those with a lower VFA. When the population was stratified according to VFA, patients with a higher VFA had a greater risk of significant hepatic steatosis, exhibited by a higher OR. The association between VFA and hepatic steatosis was independent of BMI. Moreover, VFA was predictive of hepatic steatosis not only in obese subjects but also in non-obese subjects.

Obesity is a well-known risk factor for metabolic diseases, including NAFLD. Previous studies reported that BMI is strongly correlated with fatty liver and the risk of NAFLD increases with increasing BMI [23,24]. However, there is growing evidence that visceral adipocity rather than total adipocity reflects ectopic fat accumulation. Thus, visceral adiposity is the harmful aspect of the metabolic syndrome. Although BMI and waist circumference are used as surrogate markers of obesity, they have limited ability to discriminate between visceral and subcutaneous fat compartments [25,26]. Therefore, the aim of our study was to assess the association between visceral fat and hepatic steatosis independently of total adiposity. Our results suggest that VFA is significantly associated with hepatic steatosis, whereas other obesity indicators, such as BMI, are not. This finding is in line with those of previous studies [10,27]. In our study, VFA was predictive of NAFLD irrespective of gender or obesity.

Previous studies showed a relatively low prevalence of NAFLD (6.3%-34.0%) when ultrasonography was used for diagnosis [28]. Because ultrasonography is a subjective examination that is highly dependent on the operator, and insensitive in mild (<30%) intrahepatic steatosis, the prevalence of NAFLD may have been underestimated [29]. By comparison, the CAP value, measured using TE, can detect hepatic steatosis involving as little as 10% of hepatocytes [30]. Although it is often impossible to evaluate the degree of steatosis with ultrasonography in obese subjects, TE can evaluate the degree of steatosis accurately in these patients.

Asian tends to comprise a greater proportion of non-obese patients with NAFLD than Western populations. The prevalence of NAFLD was 7.3% in a group of non-obese Chinese patients, with an incidence of 8.9% during the 5-year follow-up [31]. In Korea, the prevalence of NAFLD in non-obese subjects was 23.4%, similar to our findings [32]. Among 110 patients with a BMI <23 kg/m^2^, 22 (20.0%) had significant hepatic steatosis. The distributions of body fat and total amount of adipose tissue are important in the development of NALFD in non-obese patients [33]. Central obesity, including truncal and visceral obesity, is more strongly correlated with NAFLD development than an increase in BMI in non-obese patients [34,35]. Thus, VFA was expected to be a stronger predictor than BMI in both obese and non-obese patients. However, age and BMI were associated with NAFLD in obese Korean subjects, but not in non-obese subjects [32]. In our study, the cut-off VFA value for predicting a low risk of significant hepatic steatosis was 57.3 cm^2^ and the value for predicting a high risk was 192.2 cm^2^. In a previous Korean study, the cut-off VFA value associated with an increased risk of obesity-related disorders was 71.10 cm^2^ (sensitivity, 72.3%; specificity, 76.5%; *P* = 0.01) for patients aged 16 to 18 years [36]. The cut-off values differed according to age, but VFA consistently had a greater impact on obesity-related outcomes than did BMI.

This study had limitations. First, histological assessment was not available. Because the subjects were undergoing health check-ups, most refused a liver biopsy due to its invasiveness. The gold standard of diagnosis is still a liver biopsy, but recent studies for non-invasive markers including CAP are under way to replace liver biopsy [37]. Second, the cut-off CAP value for hepatic steatosis is controversial. In this study, the cut-off value (248 dB/m) was determined according to previous reports on the accuracy of CAP based on a meta-analysis data. However, our group has experience validating CAP scores according to histologic steatosis in patients with NAFLD [8,9]. The optimal cut-off values for steatosis were 247 dB/m for S1, 280 dB/m for S2, and 300 dB/m for S3. Third, CT scan had concerns about the cost and risk of radiation exposure. Thus, CAP is preferred due to radiation-free assessment. However, fat CT is very safe and relatively low exposure to radiation with an examination time of 3 min and a radiation exposure of only 2 mSV. It is usually half of the low-dose chest CT (5mSV) for early diagnosis of lung cancer. Lastly, sarcopenia has recently been identified as an important factor in NAFLD, but it has not been evaluated in this study.

In conclusion, VFA was significantly correlated with hepatic steatosis measured by CAP. VFA was not affected by gender or other factors. Thus, patients with NAFLD require specific surveillance, which should involve parameters indicative of central obesity and, not only BMI.

## Supporting information

S1 Fig Correlation between controlled attenuation parameter(CAP) value and visceral fat area (VFA). The regression line is shown. CAP was significantly correlated with VFA (P<0.001).
(TIF)

S1 Table Comparison of patients with and without significant hepatic steatosis subdivided by gender. (DOCX)

## Abbreviations

ALT: alanine aminotransferase
AST: aspartate aminotransferase
AUROC: areas under the receiver operating characteristic curve
BMI: body mass index
CAP: controlled attenuation parameter
CI: confidence interval
CT: computed tomography
ESR: erythrocyte sedimentation rate
IQR: interquartile range
MRI: magnetic resonance imaging
NAFLD: nonalcoholic fatty liver disease
NPV: negative predictive value
OR: odds ratio
PPV: positive predictive value
SD: standard deviation
SFA: subcutaneous fat area
TAT: total adipose tissue area
TE: transient elastography
VFA: visceral fat area
WHR: waist-hip-ratio
γ-GTP: γ-glutamyl transpeptidase
