# Supplementary figures and images for: The relationship between visceral obesity and hepatic steatosis measured by controlled attenuation parameter
Source: PLoS One. 2017 Oct 27;12(10):e0187066. doi: 10.1371/journal.pone.0187066 (PMC5659780; doi:10.1371/journal.pone.0187066)

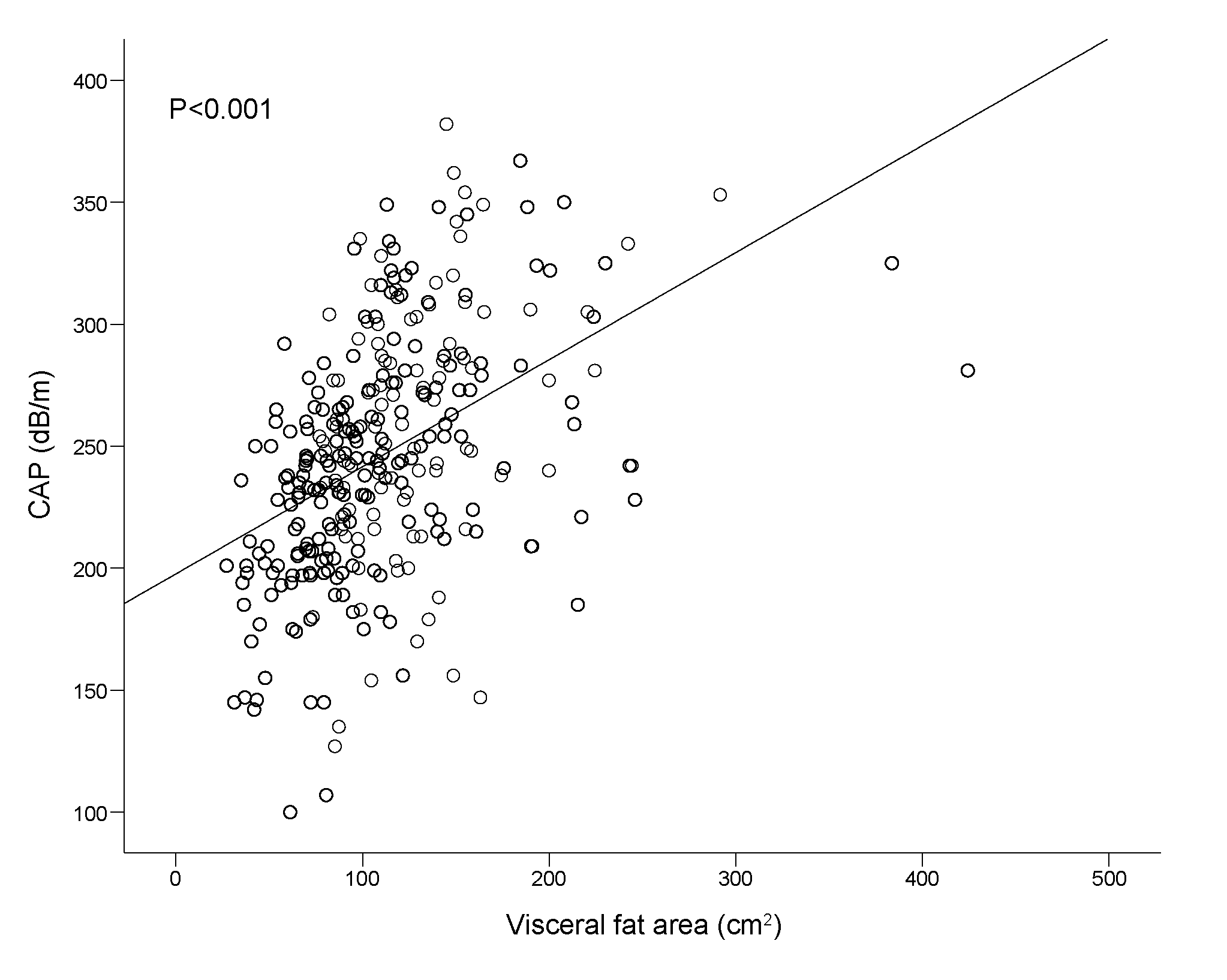

Supplement: S1 Fig — The regression line is shown. CAP was significantly correlated with VFA (P<0.001). (TIF) [file pone.0187066.s001.tif]
