# Supplementary material for: The relationship between visceral obesity and hepatic steatosis measured by controlled attenuation parameter
Source: PLoS One. 2017 Oct 27;12(10):e0187066. doi: 10.1371/journal.pone.0187066 (PMC5659780; doi:10.1371/journal.pone.0187066)
Supplement: S1 Table — (DOCX) [file pone.0187066.s002.docx]

**S1 Table.** Comparison of patients with and without significant hepatic steatosis subdivided by gender

| Variable | Male (n=165, 54.3%) | | |  | Female (n=139, 45.7%) | | | | |
| --- | --- | --- | --- | --- | --- | --- | --- | --- | --- |
|  | No significant  hepatic steatosis  (n=80) | Significant hepatic steatosis  (n=85) | *P* value |  | No significant hepatic steatosis  (n=90) | Significant hepatic steatosis  (n=49) | | *P* value | |
| Age, years | 56.8 ± 11.7 | 56.4 ± 10.2 | NS | 54.3 ± 11.1 | | 60.0 ± 8.5 | 0.002 | |  |
| Medical history |  |  |  |  | |  |  | |  |
| Diabetes mellitus | 4 (5.0) | 12 (14.1) | NS | 4 (4.5) | | 2 (4.1) | NS | |  |
| Hypertension | 17 (21.3) | 12 (14.1) | NS | 9 (10.0) | | 6 (12.2) | NS | |  |
| Body mass index, kg/m^2^ | 24.1 ± 2.6 | 26.1 ± 3.2 | <0.001 | 22.0 ± 2.3 | | 24.6 ± 2.6 | <0.001 | |  |
| Waist/Hip ratio | 0.88 ± 0.03 | 0.90 ± 0.04 | 0.001 | 0.85 ± 0.04 | | 0.88 ± 0.04 | <0.001 | |  |
| Visceral fat area, cm^2^ | 168.9 ± 68.9 | 201.9 ± 73.0 | <0.001 | 92.2 ± 47.5 | | 144.4 ± 54.6 | <0.001 | |  |
| Subcutaneous fat area, cm^2^ | 90.5 ± 40.9 | 112.0 ± 47.7 | 0.002 | 119.8 ± 46.5 | | 146.2 ± 45.8 | 0.002 | |  |
| Laboratory profiles |  |  |  |  | |  |  | |  |
| Fasting glucose, mg/mL | 100.1 ± 28.6 | 108.8 ± 26.4 | 0.043 | 91.8 ± 17.6 | | 97.8 ± 16.5 | 0.047 | |  |
| Cholesterol, mg/mL | 172.6 ± 42.3 | 183.2 ± 35.4 | NS | 190.8 ± 35.2 | | 194.7 ± 34.1 | NS | |  |
| Triglycerides, mg/mL | 112.8 ± 63.2 | 143.5 ± 80.9 | 0.008 | 88.8 ± 37.8 | | 117.1 ± 46.9 | <0.001 | |  |
| AST, IU/L | 23.6 ± 8.3 | 24.9 ± 9.3 | NS | 20.4 ± 5.7 | | 23.2 ± 10.6 | NS | |  |
| ALT, IU/L | 23.8 ± 11.5 | 29.2 ± 16.9 | 0.02 | 17.5 ± 8.2 | | 21.8 ± 13.4 | 0.018 | |  |
| γ-GTP, IU/L | 41.5 ± 35.2 | 43.7 ± 37.7 | NS | 19.4 ± 17.9 | | 26.8 ± 15.3 | 0.015 | |  |
| ESR, mm/hr | 12.4 ± 10.6 | 14.4 ± 15.4 | NS | 21.3 ± 17.2 | | 23.0 ± 16.4 | NS | |  |
| Liver stiffness value, kPa | 4.5 (2.1-21.8) | 4.6(2.8-14.3) | NS | 3.9 (2.2-25.7) | | 4.4 (2.2-16.3) | NS | |  |

Variables are expressed as mean ± standard deviation, median (range), or number (%).

NS, not significant; AST, aspartate aminotransferase; ALT, alanine aminotransferase; γ-GTP, γ-glutamyl transpeptidase; ESR, erythrocyte sedimentation rate
